# Towards Portable Large-Scale Image Processing with High-Performance Computing

**DOI:** 10.1007/s10278-018-0080-0

**Published:** 2018-05-03

**Authors:** Yuankai Huo, Justin Blaber, Stephen M. Damon, Brian D. Boyd, Shunxing Bao, Prasanna Parvathaneni, Camilo Bermudez Noguera, Shikha Chaganti, Vishwesh Nath, Jasmine M. Greer, Ilwoo Lyu, William R. French, Allen T. Newton, Baxter P. Rogers, Bennett A. Landman

**Affiliations:** 1 Electrical Engineering, Vanderbilt University, 2201 West End Ave, Nashville, TN 37235 USA; 2 Computer Science, Vanderbilt University, Nashville, TN USA; 3 Biomedical Engineering, Vanderbilt University, Nashville, TN USA; 4 Institute of Imaging Science, Vanderbilt University, Nashville, TN USA; 5 Advanced Computing Center for Research and Education, Vanderbilt University, Nashville, TN USA; 6 Radiology and Radiological Sciences, Vanderbilt University Medical Center, Nashville, TN USA; 7 Psychiatry, Vanderbilt University Medical Center, Nashville, TN USA

**Keywords:** Containerized, XNAT, DAX, VUIIS, Large-scale, Portable

## Abstract

High-throughput, large-scale medical image computing demands tight integration of high-performance computing (HPC) infrastructure for data storage, job distribution, and image processing. The Vanderbilt University Institute for Imaging Science (VUIIS) Center for Computational Imaging (CCI) has constructed a large-scale image storage and processing infrastructure that is composed of (1) a large-scale image database using the eXtensible Neuroimaging Archive Toolkit (XNAT), (2) a content-aware job scheduling platform using the Distributed Automation for XNAT pipeline automation tool (DAX), and (3) a wide variety of encapsulated image processing pipelines called “spiders.” The VUIIS CCI medical image data storage and processing infrastructure have housed and processed nearly half-million medical image volumes with Vanderbilt Advanced Computing Center for Research and Education (ACCRE), which is the HPC facility at the Vanderbilt University. The initial deployment was natively deployed (i.e., direct installations on a bare-metal server) within the ACCRE hardware and software environments, which lead to issues of portability and sustainability. First, it could be laborious to deploy the entire VUIIS CCI medical image data storage and processing infrastructure to another HPC center with varying hardware infrastructure, library availability, and software permission policies. Second, the spiders were not developed in an isolated manner, which has led to software dependency issues during system upgrades or remote software installation. To address such issues, herein, we describe recent innovations using containerization techniques with XNAT/DAX which are used to isolate the VUIIS CCI medical image data storage and processing infrastructure from the underlying hardware and software environments. The newly presented XNAT/DAX solution has the following new features: (1) multi-level portability from system level to the application level, (2) flexible and dynamic software development and expansion, and (3) scalable spider deployment compatible with HPC clusters and local workstations.

## Introduction

High-throughput medical image computing, which includes storage, processing, and analysis, is essential in exploring hidden regularities of large-scale medical image data. Efficient data storage and exchange are increasingly important for subsequent image processing. As reviewed by Izzo [1], prevalent large-scale medical imaging archiving solutions include, but are not limited, to the Extensible Neuroimaging Archive Toolkit (XNAT) [2], the eXTENsible platform for biomedical Science (XTENS) [3], and the Collaborative Informatics and Neuroimaging Suite (COINS) project [4]. Given the XNAT’s high flexibility and availability of community support, the Vanderbilt University Institute for Imaging Science (VUIIS) Center for Computational Imaging (CCI) has built a large-scale image database VUIIS XNAT [5, 6] that extends XNAT to house 379 IRB approved projects at the Vanderbilt University and Vanderbilt University Medical Center (VUMC) with 78,512 magnetic resonance imaging (MRI) or computed tomography (CT) sessions, totaling 480,574 medical imaging scans for 48,556 subjects (as of December 2017).

Large-scale medical image computing requires an efficient and stable imaging database and additionally demands efficient job management and distribution schemes to handle heterogeneous images and processing tasks (e.g., segmentation, registration, and modeling). To address such challenges, we have developed the Distributed Automation for XNAT toolkit (DAX, http://dax.readthedocs.io/en/latest/) [5, 7, 8], which is a Python-based middleware layer to control the processing algorithms for the individual scan, scan group, or session. For each image computing task, one or more image processing algorithms are encapsulated as a pipeline item (“spider”), which defines the order of executions, dependencies, and the running environment. For instance, an end-to-end multi-atlas MRI brain segmentation pipeline [9] has been wrapped as a “Multi_Atlas” spider, which contains preprocessing, N4 bias field correction [10], registrations [11], atlas selection [12], label fusion [13], and post-processing [14]. The spider defines the sequence of the processing as well as the processing environments (e.g., binary files, package dependencies, and source code). Once the spider is developed, it can be launched, managed, and terminated by DAX.

The VUIIS CCI medical image data storage and processing infrastructure (VUIIS XNAT, DAX, and spiders) have been developed in close relationship with the hardware and computing environment of the Vanderbilt Advanced Computing Center for Research and Education (ACCRE, www.accre.vanderbilt.edu) and other facilities at the Vanderbilt University. This process has ensured the effectiveness of the toolkit at the Vanderbilt University but has also exposed certain limitations. First, in 2016/2017, the Vanderbilt high-performance computing (HPC) facility decided to upgrade the underlying operating system from CENTOS 6 to CENTOS 7. Many of the VUIIS CCI image processing tools and libraries were not supported by the new platform. For instance, changes in supported versions of CUDA libraries between operating systems (OS) caused unforeseen gaps in application coverage, requiring specialized installations for various applications. This upgrade process led us to revisit the framework of the VUIIS CCI medical image storage and processing infrastructure. More generally, to deploy or migrate this infrastructure to another institution or HPC center could be laborious. Significant efforts and resources could be required to migrate the DAX platforms and spiders to a platform with differenthardware, OS, and installed packages might be very different between users and Vanderbilt University.

A second issue arose concerning spider portability. Since the initial development of DAX, individual spiders were designed to be executed across platforms (e.g., laptops, workstations, servers, and HPC nodes). The deployment worked well for several years as end users would deploy a common library of tools and then be able to use all available spiders. However, with the growth of the library, multiple releases of third-party tools, varying system level dependencies, and library incompatibilities between spiders, the processes of deploying the underlying architecture had become increasingly cumbersome and hardware/software dependent. In revisiting the framework for sustainability, we focused on improving the ease of use for the user scenario where a single spider is executed on a local laptop/workstation/server or a different HPC platform.

In this paper, we describe new innovations that create a portable solution based on the VUIIS CCI medical image data storage and processing infrastructure using virtualized VUIIS XNAT, containerized DAX, and containerized spiders to isolate the image processing platform from the underlying OS and hardware. The portable implementation is an alternative solution that enables hardware independent encapsulation and provides a convenient way to migrate our platforms to another institute or center. The containerized spiders are able to run on local workstations or alternative facilities as easily as at our local HPC center. To achieve this, the VUIIS XNAT has been deployed on a virtual machine (VM), while the containerized DAX and spiders use the Docker [15] and Singularity [16] container format. More specifically, the portable version has the following new features: (1) multi-level portability is supported, which not only allows for the deployment of the entire infrastructure (VUIIS XNAT + DAX + spiders) but also enables deploying one component (VUIIS XNAT or DAX or spiders) independently at another HPC center. (2) The containerized design allows future updates to DAX and spiders to take place without concern regarding the underlying hardware. (3) The portable feature leverages the scalability of the spiders, which are runnable on HPC clusters, workstations, and personal computers.

The remainder of the paper is organized as follows. First, XNAT and the existing VUIIS CCI medical image storage and processing infrastructure (VUIIS XNAT + DAX + spiders) are reviewed. Next, the portable solution with containerized DAX and spiders is introduced. Finally, we discuss the benefits and limitations of our implementation. This paper extends our previous descriptions [7, 8]with a more detailed analysis of the framework as well as an exposition of the new containerized spiders.

## XNAT and VUIIS XNAT

### XNAT

XNAT, developed by the Neuroinformatics Research Group at the Washington University, is an archiving platform designed for managing large-scale and multi-resource neuroimaging data [2]. XNAT serves as an interface between medical imaging equipment, image processing platforms, clinical database, and end users. Approved users can manage their medical image data through HIPPA-compliant security access, which is particularly important in medical applications. The flexibility of the XNAT framework resides in its use of XML (http://www.w3.org/XML) and XML Schema (https://www.w3.org/XML/Schema). The XML is a standard text format language designed to describe a wide variety of data formats for large-scale electronic publishing. The XNAT’s XML Schema [2, 17] supports neuroimaging data of several formats and is easily extensible by the end user for new applications.

The XNAT’s architecture is composed of a three-tier design: (1) relational database structure, (2) Java-based middleware class, and (3) user interface content. In the first tier, the relational database structure is set by a specific XML Schema Definition (XSD) [2, 17] to store non-imaging and imaging data such as Digital Imaging and Communication in Medicine (DICOM) files. Data resources are associated with Uniform Resource Identifier (URI). In the second tier, the middleware classes are established for implementing user functions upon the rational database. In the third tier, the user interface is implemented to provide two types of access: web-based interface and command line tools. The XNAT user interface enables the users to manage image processing, data access, and data distribution.

### VUIIS XNAT

The VUIIS CCI has a custom installation of XNAT, henceforth referred to as VUIIS XNAT [5, 6]. VUIIS XNAT currently stores data and processing results from 379 projects, 48,556 subjects, 78,512 imaging sessions, and 480,574 imaging scans at the Vanderbilt University (as of December 2017). To achieve scalable project management, we have built a control system for VUIIS XNAT using the Vanderbilt University’s Research Electronic Data Capture (REDCap) [18], a secure web application for building and managing online databases.

VUIIS XNAT has been designed to house data from multiple sources. Many scans come from the research MRI scanners at the VUIIS Human Imaging Core: one 7T Philips Achieva and two 3T Philips Intera/Achieva scanners. These data are routed to XNAT in DICOM format as requested by investigators and are assigned to investigator-specific projects in XNAT, leveraging the XNAT’s security controls and data sharing model to maintain study-specific data access. Additionally, DAX provides command line tools to upload data from other sources to any desired XNAT project, including support of batch uploading for large-scale imaging data. This facilitates multi-site collaborations, permitting collaborators outside of the Vanderbilt University to upload large-scale data to VUIIS XNAT with relative ease.

For most subsequent processing, DICOM images are converted to the neuroimaging community’s NIfTI-1 data format [19], which is a standard medical image format supported by most medical imaging processing software. VUIIS XNAT also contains ancillary data (if available and required by investigators) such as respiratory and cardiac data from physiological monitoring equipment. However, since VUIIS XNAT is not certified to store protected health information (PHI), all PHI is stored instead in REDCap and linked to the image data in VUIIS XNAT by numerical code to avoid potential privacy concerns.

## DAX and Spiders

### DAX

The large-scale image database is organized as different projects based on the definition by PIs. Within each project, all imaging scans and the corresponding processing results from the same patient are saved as one subject on XNAT, which might contain one session (time point) or multiple sessions. DAX was developed to provide an efficient database and processing management tool for large-scale medical image processing scenarios. It is a Python-based middleware layer used to specify the processing algorithms to apply to each image session on XNAT. First, DAX communicates with XNAT to identify which sessions match the running requirements (e.g., input files, queue permits). Then, the image processing tasks are launched for all requirement satisfied sessions based on the definition in the REDCap dashboard. To launch the processing tasks, DAX provides the scripts of image processing pipelines called spider, which defines not only the software requirements but also handles the image processing pipelines. The spiders define a list of image processing tasks and the corresponding software environments. Then, spiders are launched on ACCRE (a high-performance computing cluster housing over 6000 CPU cores) using batch processing technique. To enable the batch processing of the spiders, DAX has been developed to support the customized shell scripts using Simple Linux Utility for Resource Manangement (SLURM).

Each time a process runs, the precise version and parameters of the algorithm that ran are logged along with the processed data. Manual interaction with the data flows is possible and widely used. Once each processing job is complete, a final report is generated in Portable Document Format (PDF). The reports are tailored for each processing task and can be quickly reviewed by a human for quality assurance (QA) purposes. For security reasons, users are required to do registration on the VUIIS XNAT website (http://xnat.vanderbilt.edu/index.php/Main_Page) to use DAX. Manual interaction with the data flows is possible and widely used; in these cases, both the original automated results and the manual correction/interaction are saved.

In the currently deployed state-of-the-art DAX (version 0.7.1), the complete source code, runtime binaries libraries, and configuration files are archived in version controlled repositories linked to the high-performance computing center. Authorized users can retrieve the exact data, execution environment, and runtime arguments to recreate any analysis at a later time.

DAX has been tested on the SUN Grid Engine [20, 21], the Simple Linux Utility for Resource Management (SLURM) platform [22], and the Moab scheduler on the Terascale Open-source Resource and QUEue Manager (TORQUE) [23].

### Spiders

A spider is a processing pipeline package containing a controlling Python script, source code, and binary executable files. The controlling python script defines the running environments (e.g., input format, output format, software paths, related packages, etc.), and the execution order when more than one image processing algorithm is included in the spider. DAX, to date, supports 303 different spiders for efficient large-scale image processing. For instance, FreeSurfer brain segmentation and cortical surface extraction [24], multi-atlas segmentation [9, 13], voxel-based morphometry and other structural analysis using the SPM [25, 26], and FSL [26, 27] software suites are implemented as spiders. Also, the diffusion tensor imaging (DTI) processing and analysis spiders have been developed for quality analysis [28, 29] plus various approaches for diffusion modeling and tractography: the FSL’s BEDPOSTX [30] and TBSS [31], the Freesurfer’s TRACULA [32], and the gray matter diffusion analysis GSBSS [33]. Available cortical surface reconstruction and analysis spiders include CRUISE [34], MaCRUISE [35, 36], surface parcellation [37], and quantitative analyses on surfaces (e.g., thickness, curvatures, sulcal depth, etc.) [38, 39]. Other spiders perform lesion segmentation like TOADS [40] and Lesion Segmentation Tools (LST) [41], fMRI quality analysis [42], or abdomen segmentation using multi-atlas segmentation [14] or deep neural networks [43].

Since each update for a spider relies on different versions of source code or software packages, it is appealing to develop a spider to contain its full computing environment and be isolated from the underlying software and hardware environments.

## Portable VUIIS XNAT, DAX, and Spiders

**Fig. 1:**
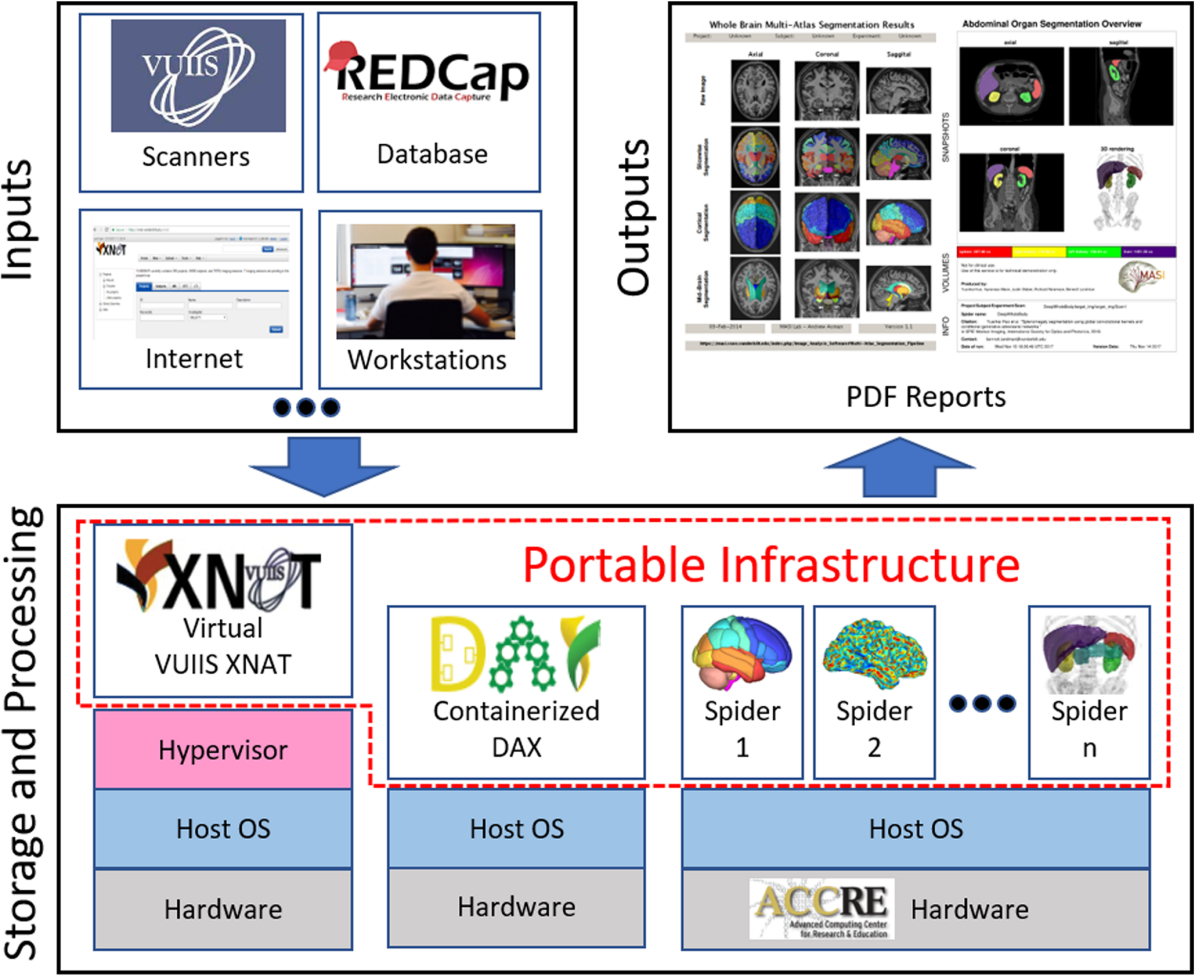
The framework of the portable implementation of VUIIS CCI medical image storage and processing infrastructure (VUIIS XNAT + DAX + spiders). The input images in VUIIS XNAT are acquired from scanners, local workstations, and Internet remote access. REDCap provides the non-imaging database as well as the processing commands to trigger the Spiders using DAX. Then, the final results are achieved as PDF format files, which are used for quality assurance purposes

The VUIIS CCI medical image storage and processing infrastructure (VUIIS XNAT + DAX + spiders) has been successfully deployed as a high-throughput large-scale medical image processing and analysis facility at the Vanderbilt University since 2011. However, since DAX and the spiders have been developed for the Vanderbilt’s specific hardware and operating system infrastructure, it can be laborious to migrate to another hardware platform or OS environment. Therefore, we now provide an additional portable implementation of the VUIIS CCI medical image storage and processing infrastructure that isolates VUIIS XNAT, DAX, and spiders from the hardware. For the portable solution, a virtual machine (VM) [44] is used to host VUIIS XNAT, while Docker [15] and Singularity [16] are used to establish containerized DAX and spiders (Fig. 1).

### VUIIS XNAT Virtual Machine

The XNAT team [2] has provided virtual machine images to run XNAT on a virtual environment built on top of the Ubuntu OS (https://wiki.xnat.org/docs16/1-installing-xnat/xnat-virtual-machine). We employ a similar approach to implement VUIIS XNAT, but we have designed our platform with two (the “dual front end” approach) virtual machines: VUIIS XNAT-I and VUIIS XNAT-II. VUIIS XNAT-I hosts web-based access, while VUIIS XNAT-II is designed for automated programmatic access and high-load command line access (e.g., DAX tools). The two virtual machine images are deployed on Hyper-V hypervisors and manage 140 terabytes (TB) Dell Network Attached Storage (NAS). A separate 30-day rolling backup is also maintained. When DAX generates a new data storage or retrieval request, the data are queued by VUIIS XNAT-II. Using this design, VUIIS XNAT is isolated from the hardware and is able to be deployed at other centers or institutes efficiently.

### Containerized DAX

Docker [15], an open-source container technology, has been increasingly successful in both industry and academia, as it provides a lightweight solution to deploy applications in an OS-independent fashion. Traditional virtual machines employ hypervisors (e.g., Hyper-V, KVM, and Xen) to emulate the virtual hardware—yet, each application requires an independent guest OS. By contrast, the Docker container rests on top of a single Linux OS instance that leads to a smaller and neater capsule containing multiple applications. Therefore, we provide the containerized DAX using a Docker container rather than VM.DAX is a lighter weight python application compared to XNAT, and with the Docker container, a working DAX does not require a full copy of an OS and all related hardware as a VM would.

The containerized DAX was built on a standard Docker container. A DockerFile was configured to set up all required packages and tools (e.g., python-dev, libxml2-dev, libxslt1-dev, wget, zip, unzip, and zlib1g-dev). The DockerFile for containerized DAX is available on the DAX Github (https://github.com/VUIIS/dax). The containerized DAX was designed for managing containerized Spiders.

A significant concern when using Docker containers on HPC clusters is security, as Docker processes run with root level privileges (such as user escalation). Singularity [16] is a new technique to support container execution without root privileges. Moreover, users are able to establish a Singularity container directly from a Docker container using the “singularity import” tool. Therefore, we also provide a Singularity DAX container that runs the processing in user space for the scenarios that root privileges are not permitted or not preferred. As a result, the OS of the image processing software is now separate from the underlying cluster, which means that the exact computational environment can be easily reproduced anywhere Singularity is installed. The containerized DAX aids reproducibility and decouples the DAX workflow from the software libraries (including the specific versions) installed on a computational system.

### Containerized Spiders

**Fig. 2:**
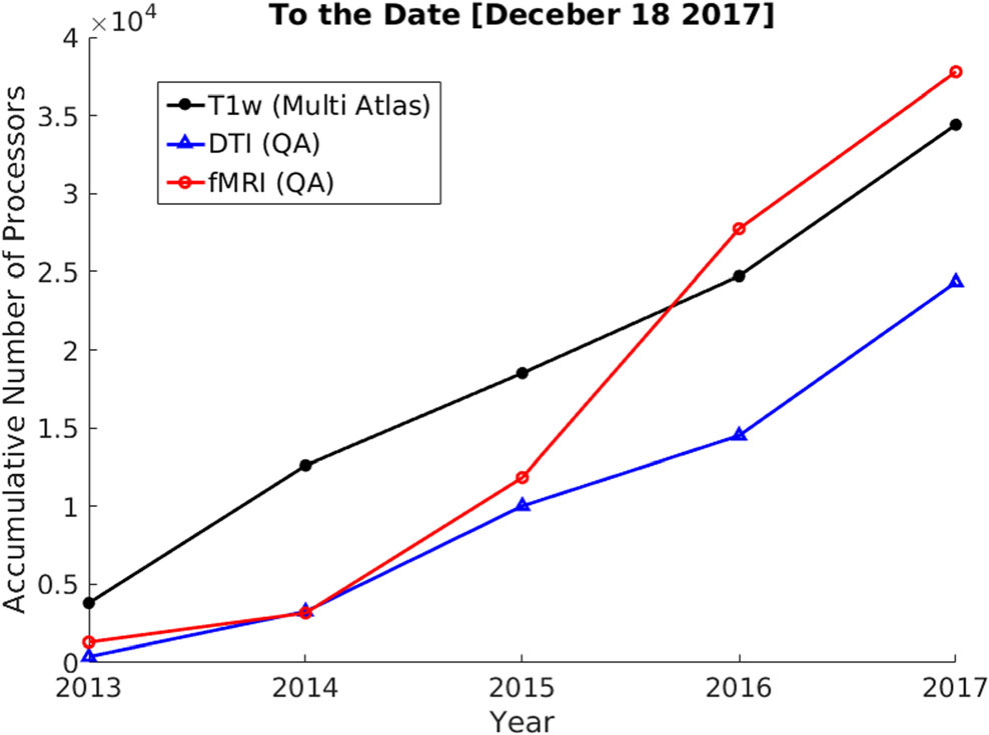
This figure presents the change in a total number of processors for Multi_Atlas, DTIQA, and fMRIQA pipelines between the year 2013 and 2017

The total number of scans that have been processed by spiders has been increasing steadily using VUIIS CCI infrastructure. Figure 2 demonstrated the number of Multi_Atlas, DTI_QA, and fMRI_QA spider processing tasks that have been computed in the past 5 years. Each spider called by DAX is an application that performs one or more medical image processing tasks. However, since the spiders were designed for the Vanderbilt University HPC infrastructure, it was difficult to run on other HPC clusters or local workstations directly unless the same OS and packages were already installed. To enable the portable deployment of a spider, we encapsulate the corresponding OS environment, software packages and source code to a Docker container.

So far, we have containerized five representative spiders from the Medical-image Analysis and Statistical Interpretation (MASI) Lab: (1) whole brain multi-atlas segmentation spider (“Multi_atlas”), (2) cortical surface reconstruction spider (“MaCRUISE”), (3) cortical surface parcellation pipeline (“Surface_Parcel”), (4) spleen segmentation pipeline (“Deep_Spleen”), and (5) multi-organ segmentation pipeline (“Deep_MultiOrganSeg”). Briefly, the “Mulit_Atlas” spider performs multi-atlas segmentation pipeline using Non-Local Spatial Staple (NLSS) label fusion technique. [9, 13]. The “MaCRUISE” spider performs consistent whole brain segmentation and cortical surface reconstruction. [35, 36]. The “Surface_Parcel” spider parcellate the whole brain cortical surfaces to 98 regions of interests (ROIs) [37]. The “Deep_SpleenSeg” spider performs spleen segmentation for both normal and splenomegaly patients on clinically acquired CT scans [43]. The “Deep_MultiOrganSeg” spider performs segmentation for liver, left kidney, right kidney, spleen, and stomach for normal patients on clinically acquired CT scans [43].

**Fig. 3:**
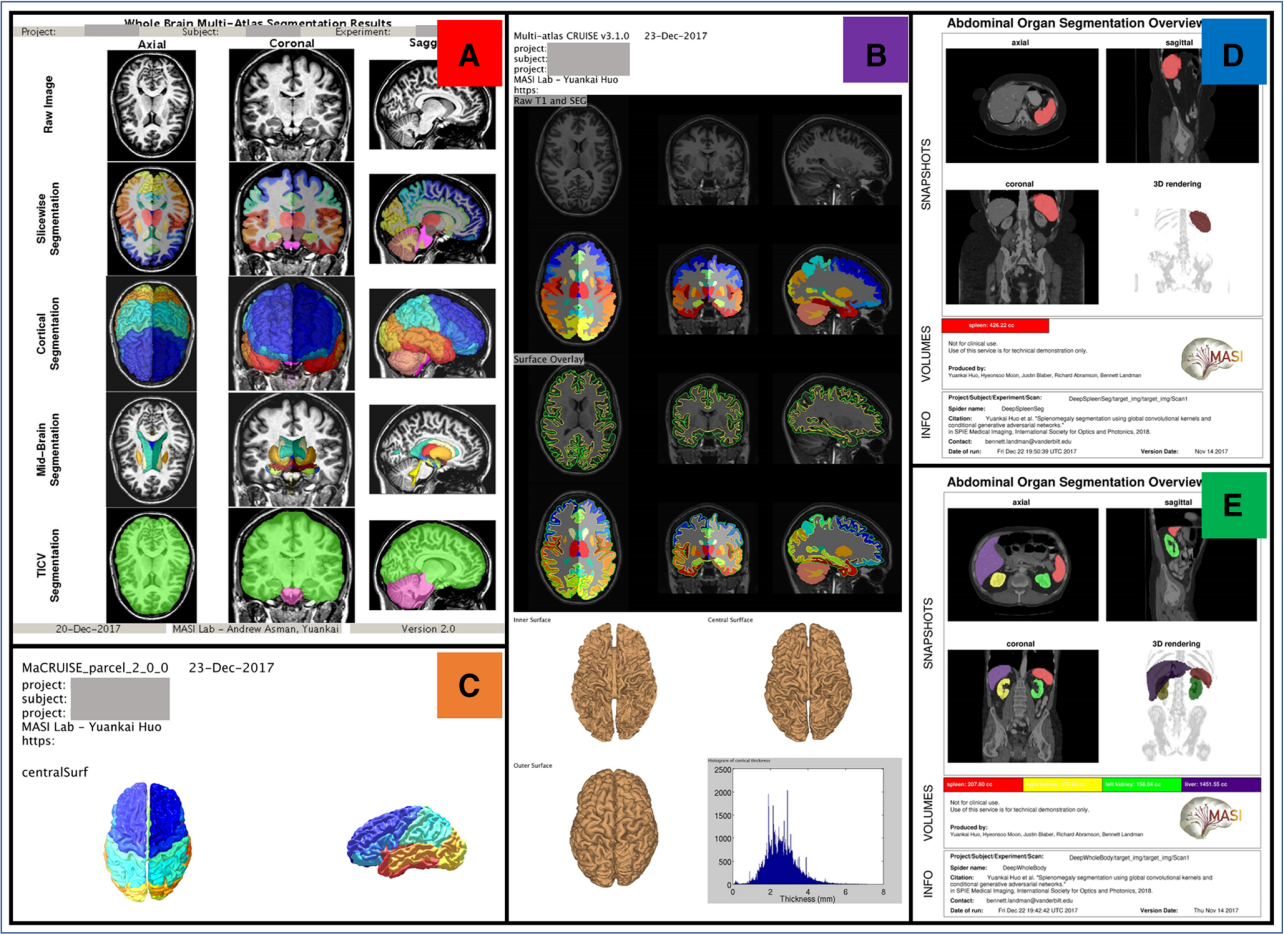
The qualitative analysis reports of the representative containerized spiders including “Multi_Atlas” (**a**), “MaCRUISE” (**b**), “Surface_Parcel” (**c**), “Deep_SpleenSeg” (**d**), and “Deep_MultiOrganSeg” (**e**).

The final PDF reports generated from the spiders are presented in Fig. 3. The OS for Docker containerized spiders is Ubuntu 16.04 LTS and Centos 6. The first three Docker containers are designed for CPU usage, while the latter two are compatible with both CPU and GPU computing. To enable GPU acceleration, we used the NVIDIA-Docker.

### Usage Example

**Fig. 4:**
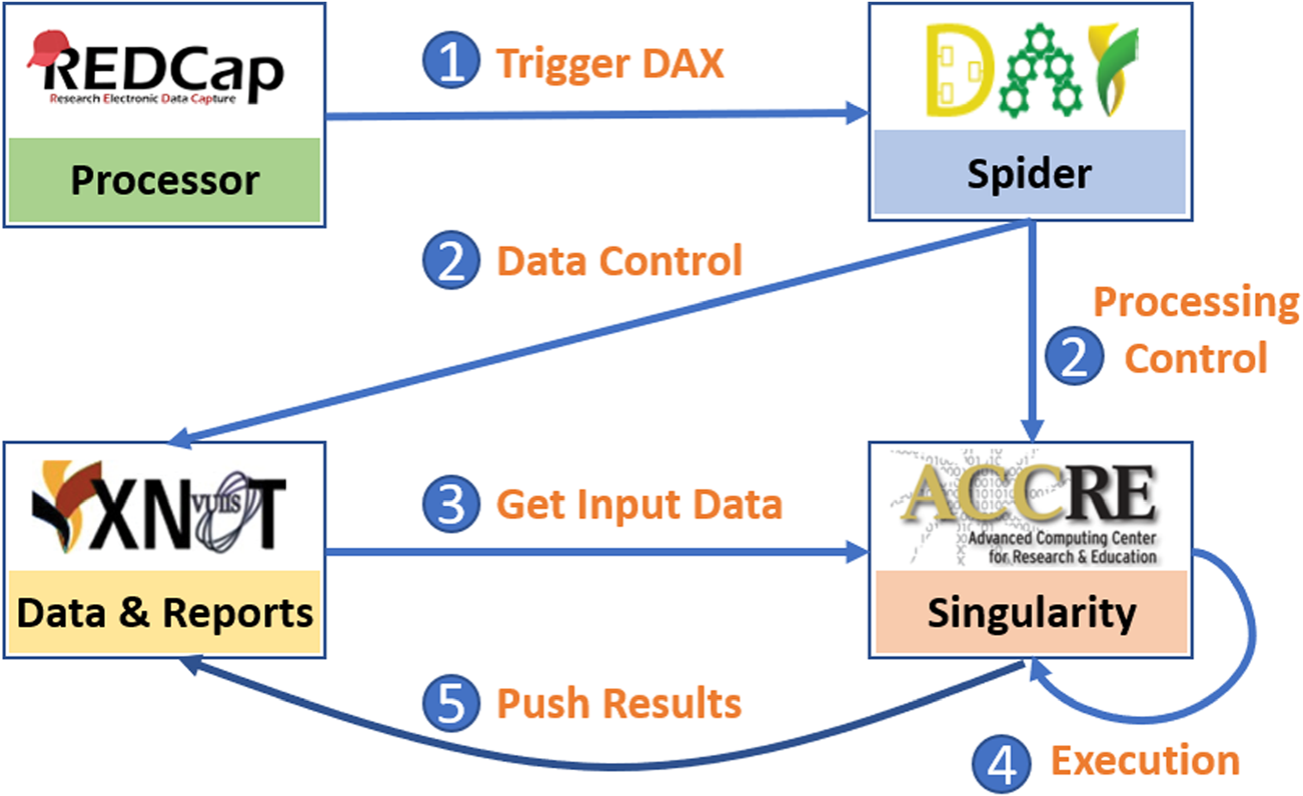
The working flow of image processing tasks using VUIIS CCI medical image data storage and processing infrastructure. (1) RedCap triggers DAX to start image processing based on a pre-defined “.yaml” configuration file, specifying which spider to execute (code is available on https://github.com/MASILab/Containerized_DAX). (2) DAX defines data retrieval, computing environment, and software dependencies from a python based spider (code is provided in APPENDIX). (3) Computational infrastructure (e.g., ACCRE) downloads the input data from XNAT. (4) Computational infrastructure executes the related processing algorithms (e.g., “Deep_MultiOrganSeg,” whose Docker image is available on https://hub.docker.com/r/masidocker/spiders/). (5) The results (e.g., segmentation volumes and final report in Fig. 3e) are pushed back to XNAT database

An example of a containerized spider is presented with python source code (in APPENDIX). A test CT abdomen scan, from the MICCAI 2015 challenge “Multi-atlas labeling beyond the cranial vault—workshop and challenge,” is provided as an example input. The related source code has been made open-source online (https://github.com/MASILab/Containerized_DAX), while the corresponding Docker/singularity image has been provided publically available (https://hub.docker.com/r/masidocker/spiders/). The processing workflow is illustrated in Fig. 4, and the final results are shown as a PDF report in Fig. 3e.

## Discussion

In summary, all human research scans taken at VUIIS today are automatically routed to a long-term PACS archive and mirrored in an XNAT server to provide secure, multi-site access to data resources. Our VUIIS XNAT/DAX systems have enabled large-scale automated analysis and quality assurance/control with a reasonable level of human oversight. Since 2010, DAX/XNAT has used 650+ CPU-years (5.6M+ CPU-hours) of image analysis at ACCRE and 200+ CPU-years (1.8M+ CPU-hours) on the MASI grid. To improve portability of the VUIIS CCI medical image storage and processing infrastructure (VUIIS XNAT + DAX + spiders), the new innovations provide an additional implementation that isolates the infrastructure from the underlying hardware and OS. The portable infrastructure may be deployed to another HPC center conveniently. Moreover, the containerized spiders are ready to run on HPC clusters or workstations, which enables efficient algorithm deployment and version management.

Although large-scale image processing has become essential in scientific research and clinical investigation, very few modern approaches are routinely used in clinical practice. A key barrier to clinical translation of image processing techniques is the evaluation and characterization of techniques at scale. Existing medical imaging automation and data interaction approaches have largely been designed for traditional well-controlled studies, and it is difficult to apply these frameworks to the more diverse data of clinical practice. With “big” imaging data, acquisitions are not uniform (e.g., multi-site issues, hardware/software changes, acquisition differences, personnel changes, etc.), and it is not clear which of the myriad of potential factors will be most challenging for image processors to generalize across. Furthermore, as many medical imaging applications begin to utilize deep learning approaches, accessible and translatable access to GPU interfaces and hardware is paramount for broad usage. Hence, creating algorithms to robustly function with big imaging data becomes a Herculean effort, in particular in the context of GPU programming.

Another interesting direction is to further improve the efficiency of large-scale data storage and image processing. We have previously investigated the theory, algorithm, and implementation of using Hadoop [45] and a data colocation grid framework [46]. We also presented a “medical image processing-as-a-service” grid framework that offers promise in utilizing the Apache Hadoop ecosystem and HBase distributed data store for data colocation by moving computation close to medical image storage [47]. It is appealing to integrate such techniques with the Vanderbilt University HPC clusters to improve computational efficiency under Big Data scenarios.

## Appendix

This appendix presents an example spider (“Deep_MultiOrganSeg”), which executes the multi-organ deep learning based segmentation. The related source code and illustrations have been made freely publically available on https://github.com/MASILab/Containerized_DAX.
